# Astrocytes regulate the balance between plasminogen activation and plasmin clearance via cell-surface actin

**DOI:** 10.1038/celldisc.2017.1

**Published:** 2017-02-21

**Authors:** Aurélien Briens, Isabelle Bardou, Héloïse Lebas, Lindsey A Miles, Robert J Parmer, Denis Vivien, Fabian Docagne

**Affiliations:** 1 INSERM/University of Caen Normandie, INSERM U1237, GIP Cyceron, Physiopathology and Imaging of Neurological Disorders (PhIND), Caen, France; 2 Department of Cell and Molecular Biology, The Scripps Research Institute, La Jolla, CA, USA; 3 Department of Medicine, University of California San Diego, La Jolla, CA, USA; 4 CHU Caen, Department of Clinical Research, CHU Côte de Nacre, Caen, France

**Keywords:** astrocytes, BDNF, endocytosis, fibrinolysis, plasminogen

## Abstract

Plasminogen activation is involved in many processes within the central nervous system, including synaptic plasticity, neuroinflammation and neurodegeneration. However, the mechanisms that regulate plasminogen activation in the brain still remain unknown. Here we demonstrate that astrocytes participate in this regulation by two mechanisms. First, the astrocyte plasma membrane serves as a surface for plasminogen activation by tissue-type plasminogen activator. This activation triggers downstream plasmin-dependent processes with important impacts in brain health and disease, such as fibrinolysis and brain-derived neurotrophic factor conversion. Second, astrocytes take up plasminogen and plasmin in a regulated manner through a novel mechanism involving endocytosis mediated by cell-surface actin and triggered by extracellular plasmin activity at the surface of astrocytes. Following endocytosis, plasminogen and plasmin are targeted to lysosomes for degradation. Thus, cell-surface actin acts as a sensor of plasmin activity to induce a negative feedback through plasmin endocytosis. This study provides evidence that astrocytes control the balance between plasmin formation and plasmin elimination in the brain parenchyma.

## Introduction

Plasminogen activation system refers to the enzymatic processes leading to regulated activation of the zymogen plasminogen into the broad-spectrum serine protease plasmin. This system was initially described in the vasculature, where it regulates fibrinolysis (the degradation of fibrin clots). In addition to this, the plasminogen activation system is also found within the central nervous system, where it controls crucial pathological and physiological processes. Indeed, under physiological conditions, plasminogen activation promotes brain-derived neurotrophic factor (BDNF) maturation, contributing to synaptic plasticity [1]. In neuroinflammatory conditions, impaired plasminogen activation is responsible for intracerebral fibrin accumulation and subsequent axonal degeneration [2, 3].

While these previous studies have focused on the characterization of plasminogen activators in the central nervous system, so far, only limited data are available regarding how plasminogen activation and plasmin activity are regulated in the central nervous system. In particular, it is now well established that plasminogen needs to bind either to a cell surface, to extracellular proteins such as fibrin or to non-fibrin cofactors for efficient activation [4, 5]. However, in the brain parenchyma, the cell type(s) responsible for stimulating this activation still remain unknown. Also, once plasmin is generated, due to its wide range of action, regulatory mechanisms are required to restrict its activity to a very close spatiotemporal window. This is why the issue of cerebral plasmin clearance systems needs to be addressed.

Astrocytes are involved in many important processes in the central nervous system, such as synaptic transmission, synapse formation and plasticity, blood–brain barrier maintenance, neurotoxicity and nervous system repair [6]. All these functions are, at least in part, related to the ability of astrocytes to control the composition of the local neuronal environment through processes of uptake and release. Interestingly, it has been shown that astrocytes are key regulators of plasminogen activators [7] and of plasmin substrates [8] through specific receptor-mediated endocytosis. However, to date, the ability of astrocytes to control plasminogen activation and plasmin activity has not been investigated.

Here we provide compelling evidence that astrocytes serve as a surface for plasminogen activation by tissue-plasminogen activator (tPA) and that this property stimulates pro-BDNF activation and fibrinogen degradation. We show that astrocytes take up plasminogen and plasmin through an endocytotic process mediated by cell-surface actin and triggered by extracellular plasmin activity at the surface of astrocytes. Therefore, cell-surface actin should be considered as a sensor for plasmin activity at the cell surface of astrocytes, engaging endocytotic processes. Following endocytosis, plasminogen and plasmin are targeted to lysosomes for degradation. This study identifies astrocytes as a cell type responsible for activation of plasminogen and clearance of plasmin.

## Results

### Astrocytes serve as a surface for plasminogen activation and subsequent plasmin-dependent proteolysis

**Figure 1:**
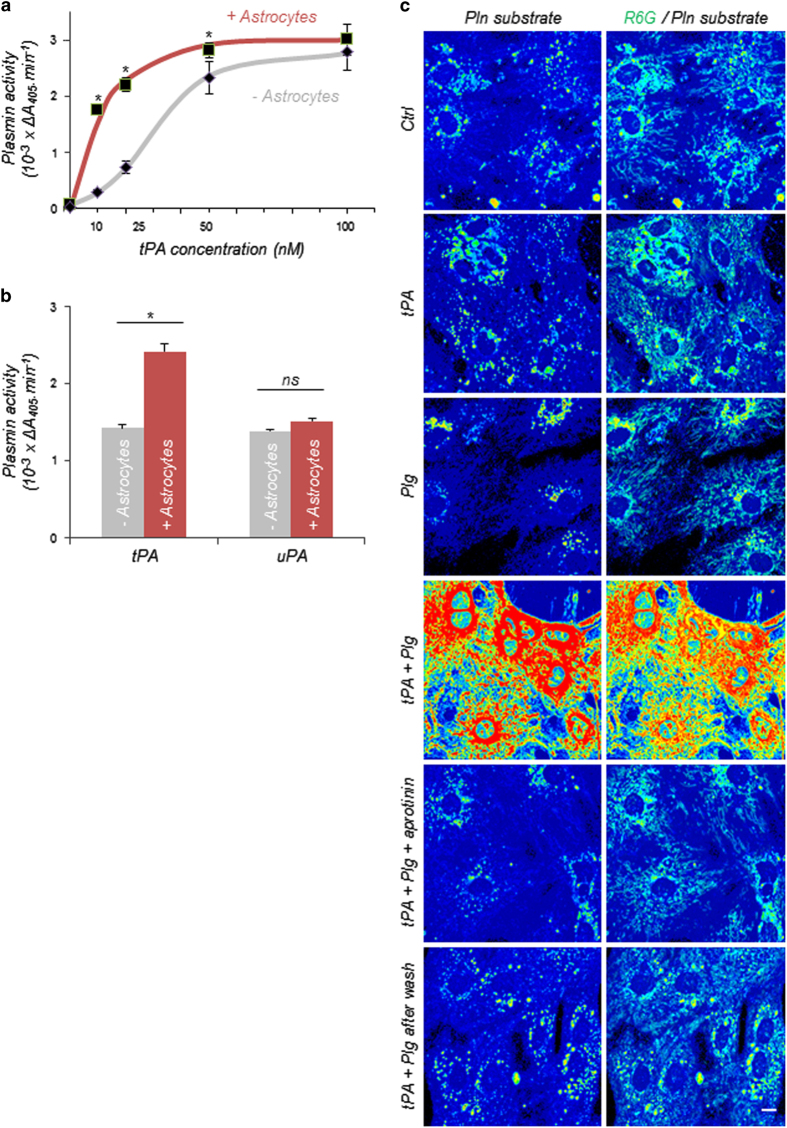
Astrocytes serve as a surface for plasminogen (Plg) activation. (**a**) Plasmin (Pln) activity (variation of absorbance of S-2251 at 405 nm, 10^−3^×Δ*A*_405_ min^−1^) was monitored for 4 h with our quantitative enzymatic assay in batch (grey) or on cultured astrocytes (red) during incubation of Plg (50 nm) with increasing doses of tPA (10–100 nm). Graph show means±s.e.m. (*n*=3). *Significantly different from ‘batch’ condition (*P*<0.05). (**b**) Pln activity (variation of absorbance of S-2251 at 405 nm, 10^−3^×Δ*A*_405_ min^−1^) was monitored with our quantitative enzymatic assay in batch (grey) or in the bathing medium of astrocytes (red) after 1 h incubation of Plg (50 nm) with tPA (25 nm) or uPA (1 UI ml^−1^, equivalent to tPA activity at 25 nm). Graph show means±s.e.m. (*n*=3). *Significantly different from ‘batch’ condition (*P*<0.05). (**c**) Representative confocal images of astrocytes (labelled with R6G, in green) incubated with a specific Pln fluorescent substrate (Sensolyte AFC Plasmin Activity Assay Kit, 10 μm) showing Pln activity at the surface of astrocytes incubated with tPA (25 nm) and Plg (50 nm) for 1 h. The substrate staining disappears in the presence of the Pln inhibitor aprotinin (20 IU ml^−1^) or after extensive wash (*n*=3). Scale bar: 20 μm. NS, nonsignificant.

To investigate the role of astrocytes in the plasminogen activation system, we first used a quantitative plasmin enzymatic assay to compare plasminogen activation by tPA in the presence of primary astrocytes or in cell-free culture medium (batch). This method, by assessing plasmin activity, gives an indirect measurement of plasminogen activation by tPA and plasmin formation. We observed that the presence of astrocytes stimulates plasminogen activation by tPA, especially at low tPA concentrations (10 or 25 nm), suggesting that astrocytes increase plasminogen accessibility for activation by tPA (half-maximal effective concentration: 36 nm in the absence of astrocytes vs 12 nm in the presence of astrocytes) (Figure 1a). We therefore hypothesized that astrocytes could represent a surface for plasminogen activation by presenting specific tPA- and plasminogen-binding molecules, thus favouring their interaction. To confirm this hypothesis, we compared the effect of astrocytes on plasminogen activation mediated by tPA and urokinase-type plasminogen activator (uPA). We observed that plasmin formation was enhanced by astrocytes only when plasminogen was incubated with tPA but not when incubated with uPA (Figure 1b). Finally, to confirm the role of astrocytes as a plasminogen activation surface, we imaged plasmin activity by confocal microscopy, using a specific fluorogenic plasmin substrate. When living astrocytes (stained by the cell-permeant dye rhodamine 6G (R6G)) are coincubated with tPA and plasminogen, a strong plasmin activity is detected at the surface of astrocytes (Figure 1c). Loss of detection of plasmin activity in the presence of the plasmin inhibitor aprotinin confirmed plasmin substrate specificity (Figure 1c). Removal of extracellular molecules after the addition of tPA and plasminogen, through a previously described method based on extensive washes of astrocytes [9], led to a complete loss of plasmin activity in cultured astrocytes, suggesting that plasminogen activation by tPA occurs at the surface of astrocytes (Figure 1c).

**Figure 2:**
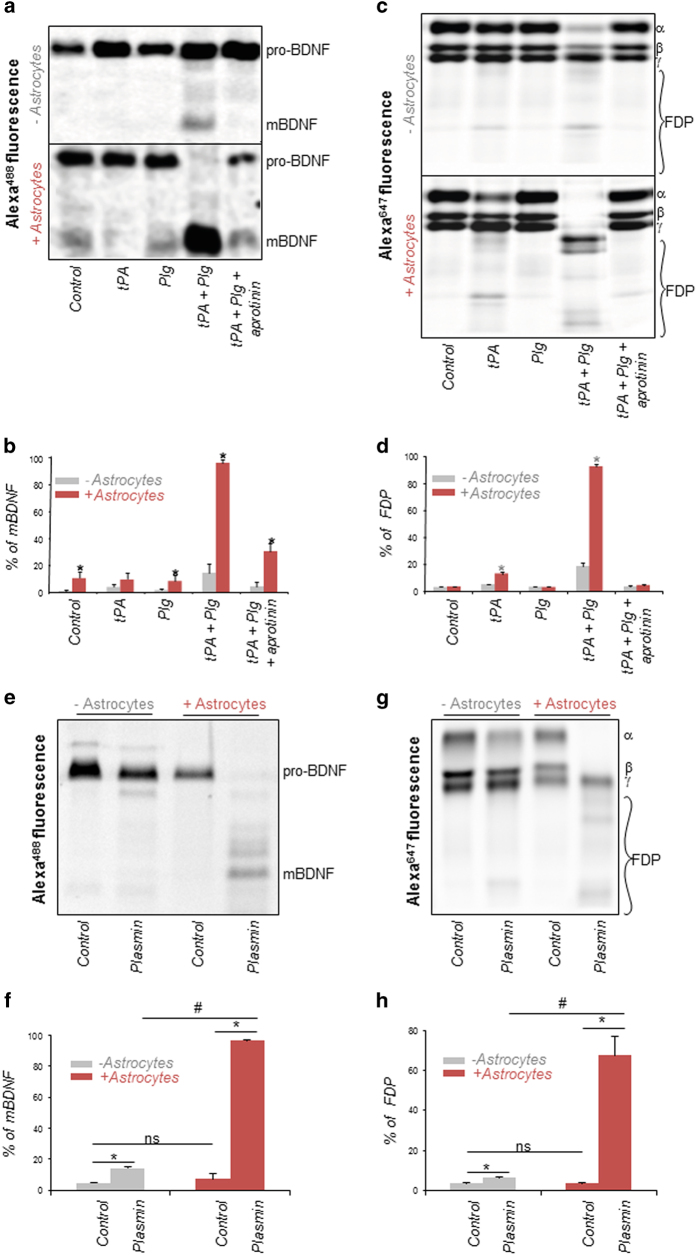
Astrocytes stimulate plasminogen (Plg) activation and act as cofactors of plasmin (Pln) activity to enhance BDNF conversion and fibrinogen degradation. (**a**) Proteins from culture supernatants of astrocytes or from batch (− astrocytes) incubated with fluorescent pro-BDNF^488^ (100 nm) in control conditions or with tPA (10 nm), Plg (50 nm), tPA+Plg (10 nm; 50 nm) or tPA+Plg+aprotinin (10 nm; 50 nm; 20 UI ml^−1^, respectively) for 1 h were submitted to SDS-PAGE. Revelation of Alexa^488^ fluorescence in the gel allowed then to distinguish the pro-BDNF form from its mature form (mBDNF). (**b**) Densitometry of SDS-PAGE bands for pro-BDNF/mBDNF ratio in the indicated conditions. Graph show means±s.e.m. (*n*=3). *Significantly different from ‘batch’ condition (*P*<0.05). (**c**) Proteins from bathing medium of astrocytes or from batch incubated with fluorescent fibrinogen^647^ (100 nm) in control conditions or with tPA (10 nm), Plg (50 nm), tPA+Plg (10 nM, 50 nm) or tPA+Plg+aprotinin (10 nm; 50 nm; 20 UI ml^−1^, respectively) for 1 h were subjected to SDS-PAGE. Revelation of Alexa^647^ fluorescence in the gel allowed then to distinguish the fibrinogen form from FDPs. (**d**) Densitometry of SDS-PAGE bands for fibrinogen/FDP ratio in the indicated conditions. Graphs show means±s.e.m. (*n*=3). *Significantly different from ‘batch’ condition (*P*<0.05). (**e**) Proteins from culture supernatants of astrocytes (+astrocytes) or from batch (−astrocytes) incubated with fluorescent pro-BDNF^488^ (100 nm) in the absence (Control) or in the presence of recombinant Pln (25 nm) for 1 h were submitted to SDS-PAGE. Revelation of Alexa^488^ fluorescence in the gel allowed then to distinguish the pro-BDNF form from its mature form (mBDNF). (**f**) Densitometry of SDS-PAGE bands for pro-BDNF/mBDNF ratio in the indicated conditions. Graphs show means±s.e.m. (*n*=3). *Significantly different from ‘Control’ condition (*P*<0.05). (**g**) Proteins from bathing medium of astrocytes or from batch incubated with fluorescent fibrinogen^647^ (100 nm) in the absence (Control) or in the presence of recombinant Pln (25 nm) for 1 h were submitted to SDS-PAGE. Revelation of Alexa^647^ fluorescence in the gel allowed then to distinguish the fibrinogen form from FDPs. (**h**) Densitometry of SDS-PAGE bands for fibrinogen/FDP ratio in the indicated conditions. Graph show means±s.e.m. (*n*=3). *Significantly different from ‘Control’ condition (*P*<0.05). NS, nonsignificant.

Plasminogen activation within central nervous system tissues has distinct roles through plasmin-dependent processing of different substrates: Under physiological conditions, the plasminogen activation system stimulates neuronal plasticity by supporting pro-BDNF maturation into its mature form (mBDNF) [1]; Under pathological conditions, plasminogen activation contributes to neuroprotection through plasmin-mediated removal of intraparenchymal fibrinogen/fibrin deposits [2]. We thus addressed whether astrocyte-driven plasminogen activation could lead to conversion of pro-BDNF into mBDNF and/or to enhanced fibrinolysis. To this end, we first compared the efficiency of conversion of Alexa^488^-labelled pro-BDNF (pro-BDNF^488^) when incubated with tPA and plasminogen in the presence or in the absence of astrocytes. Electrophoretic profiles of the pro-BDNF and mBDNF forms (Figure 2a) and corresponding quantification (Figure 2b) revealed that astrocytes stimulate BDNF maturation when the latter is coincubated with tPA and plasminogen (14% of mBDNF in the absence of astrocytes vs 95% in the presence of astrocytes). In the same way, we studied electrophoretic profile of Alexa^647^-labelled fibrinogen incubated with tPA and plasminogen (alone or in combination) in the presence or in the absence of astrocytes (Figure 2c). Quantification of the proportion of fibrinogen and its degradation products (FDPs) (Figure 2d) revealed that astrocytes stimulate fibrinogen degradation when coincubated with tPA and plasminogen. Blockade of pro-BDNF conversion and fibrinogen degradation by the plasmin inhibitor aprotinin confirmed the role of plasmin formation in these experiments (Figure 2a–d). We then wondered if astrocytes could stimulate plasmin activity, thus acting as a cofactor for plasmin in the processing of its substrates. To answer this question, we compared plasmin-induced cleavage of pro-BDNF and fibrinogen in the presence or in the absence of astrocytes. We observed that pro-BDNF cleavage (Figure 2e and corresponding quantification, Figure 2f) and fibrinogen degradation (Figure 2g and corresponding quantification, Figure 2h) were both increased in the presence of astrocytes. Altogether, these data indicate that astrocytes, by facilitating the activation of plasminogen by tPA at their surface and by acting as a cofactor for plasmin, drive the proteolytic processing of its substrates, such as pro-BDNF or fibrinogen.

### Astrocytes drive the endocytosis of plasminogen and plasmin, a process activated by plasmin activity

One of the main functions of astrocytes is to control extracellular compartment composition through processes of internalization. Interestingly, astrocytes regulate the levels of the plasminogen activator tPA [7] and the plasmin substrate pro-BDNF [8] by internalizing them. However, so far, the possibility that astrocytes could regulate the plasminogen activation system by internalizing plasminogen and/or plasmin was never investigated.

**Figure 3:**
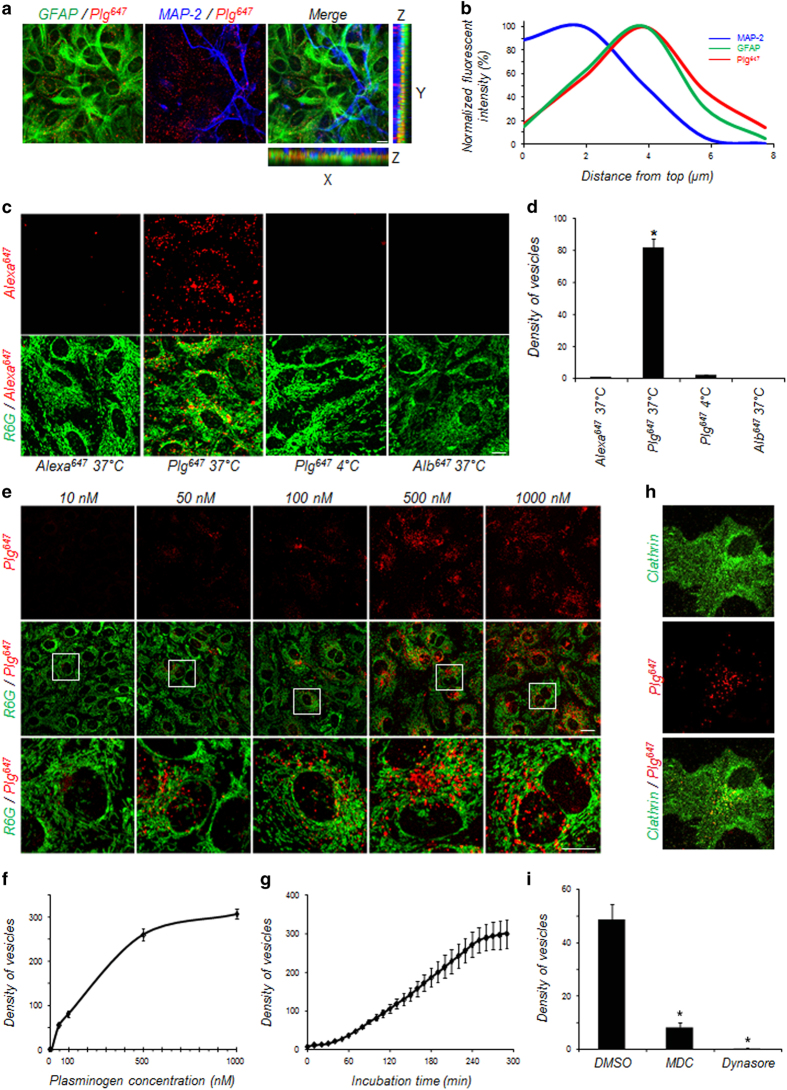
Astrocytes drive plasminogen (Plg) endocytosis. (**a**) Representative photomicrograph shows fluorescence after immunocytochemistry of neurons (MAP-2, blue) and astrocytes (glial fibrillary acidic protein (GFAP, green) performed on mixed cultures treated with fluorescent Alexa^647^-labelled Plg (Plg^647^, 25 nm, red; *n*=3) for 1 h. (**b**) Representative fluorescent intensity—distance graph measured from confocal images in (**a**) showing that extracellular Plg (Plg^647^) accumulates almost exclusively in astrocytes (*n*=3). (**c**) Representative confocal images of cultured astrocytes (R6G, green) exposed to Alexa^647^ (100 nm), Alexa^647^-labelled albumin (Alb^647^, 25 nm) or Plg^647^ (25 nm) at 37 °C or Plg^647^ (25 nm) at 4 °C for 1 h (*n*=5). (**d**) Quantification of fluorescent vesicles (number of vesicles/10^3 ^μm^3^, *n*=4) in astrocytes incubated with Alexa^647^, Alb^647^ or Plg^647^ at 37 °C or Plg^647^ at 4 °C for 1 h (*n*=5). *Significantly different from ‘Alexa^647^ 37 °C’ condition (*P*<0.05). (**e**) Representative confocal images of cultured astrocytes (R6G, green) exposed for 1 h to increasing doses (10–1 000 nm) of Plg^647^ and (**f**) corresponding quantification (number of vesicles/10^3^ μm^3^, *n*=4) shows dose-dependent and time-dependent (**g**) uptake of Plg^647^ by astrocytes. (**h**) Representative photomicrograph shows fluorescence after immunocytochemistry for clathrin (green) in cultured astrocytes treated with Plg^647^ (25 nm, red) for 1 h. (**i**) Quantification of Plg^647^-positive vesicles (number of vesicles/10^3 ^μm^3^, *n*=4) in astrocytes treated for 1 h with the inhibitor of clathrin-mediated endocytosis monodansylcadaverine (MDC; 100 μm) or with the dynamin inhibitor dynasore (50 μm). Graphs show means±s.e.m. (*n*=4). *Significantly different from dimethyl sulfoxide (DMSO) condition (*P*<0.05). Scale bars: 20 μm.

To follow plasminogen fate, we used recombinant Alexa^647^-labelled plasminogen (Plg^647^). After incubation in mixed cultures of neurons and astrocytes, we observed a punctate fluorescent signal due to Plg^647^ within the cytoplasm of astrocytes but not within neurons (Figure 3a and b). We next decided to characterize this phenomenon in pure astrocyte cultures. We first confirmed that this process is specific for plasminogen as Alexa^647^ alone or albumin labelled with Alexa^647^ (Alb^647^) did not accumulate within astrocytes (Figure 3c and d). In addition, the absence of Plg^647^ detection in astrocytes at 4 °C is consistent with an active process of internalization (Figure 3c and d). This internalization occurred in a dose-dependent (from 0 to 1 μm; Figure 3e and f) and time-dependent (from 10 to 300 min, Figure 3g) manner. Altogether, these data suggest that the internalization of plasminogen into astrocytes is achieved through active endocytosis.

As a first proof of endocytosis-mediated uptake of exogenous tPA, we showed that intracellular Plg^647^ colocalizes in glial fibrillary acidic protein (GFAP)-positive astrocytes with clathrin (Figure 3h), a protein having a crucial role in plasma membrane invagination necessary for the formation of endocytosis vesicles. Pre-treatment of cells in the presence of monodansylcadaverin, to promote disassembly of clathrin cages, or dynasore, an inhibitor of dynamin, reduced the density of vesicles containing Plg^647^ in GFAP-positive astrocytes (Figure 3i). These data indicate that astrocytes uptake plasminogen through an active and specific endocytosis process mediated by clathrin and dynamin.

**Figure 4:**
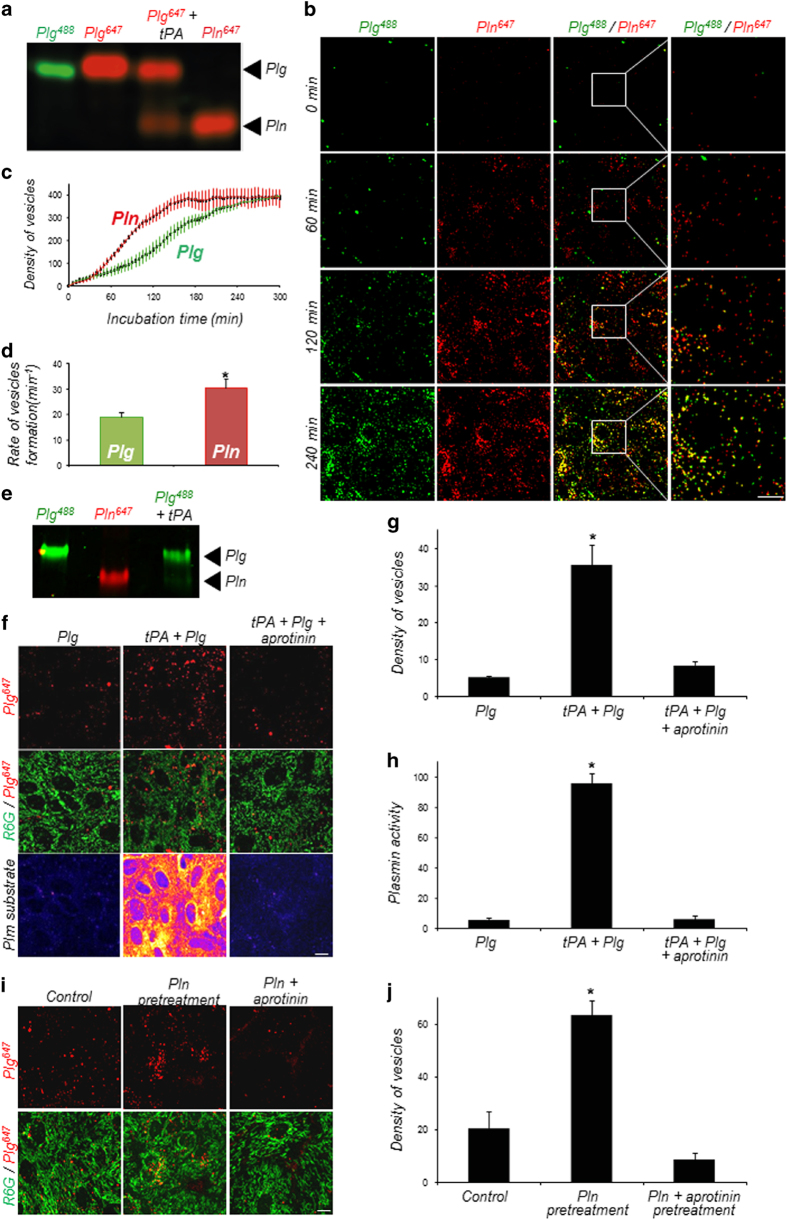
A plasmin (Pln) substrate is involved in plasminogen (Plg) and Pln uptake. (**a**) Electrophoretic analysis showing Plg and Pln forms present in the cell monolayer of astrocytes incubated with Alexa^488^-labelled Plg (Plg^488^), Alexa^647^-labelled Plg (Plg^647^), Alexa^647^-labelled Plg and tPA (Plg^647^+tPA, 25 nm) or Alexa^647^-labelled Pln (Pln^647^) for 1 h (*n*=3). (**b**) Time course (0–240 min) of Plg^647^ (25 nm, green) and Pln^647^ (25 nm, red) uptake in cultured astrocytes (*n*=3). (**c**) Quantification (number of vesicles/10^3^ μm^3^, *n*=4) of Plg^488^ and Pln^647^ uptake by cultured astrocytes as a function of time (0–300 min) and (**d**) corresponding quantification of uptake kinetics (rate of vesicles formation per min). Graphs show means±s.e.m. (*n*=3). *Significantly different from ‘Plg’ condition (*P*<0.05). (**e**) Cultured astrocytes were treated with Plg^488^ (25 nm), Pln^647^ (25 nm) or Plg^488^ (25 nm) with recombinant tPA (10 nm) for 2 h. Then, proteins were removed with a specific washing protocol and intracellular proteins were extracted and submitted to SDS-PAGE. Alexa^488^ and Alexa^647^ fluorescence was then revealed in the gel allowing to distinguish between the Plg and the Pln forms. (**f**) Representative confocal images of cultured astrocytes (R6G, green) exposed to Plg^647^ (25 nm, red) with or without tPA (25 nm; tPA+Plg) or a combination of tPA and aprotinin (20 IU ml^−1^; tPA+Plg+aprotinin) for 1 h. A specific fluorescent Pln substrate was coincubated in the medium, to monitor Pln activity (*n*=4). (**g**) Corresponding quantification of density (number of vesicles/10^3 ^μm^3^) of fluorescent vesicles and (**h**) Pln substrate fluorescence intensity (arbitrary units). Graphs show means±s.e.m. (*n*=4). *Significantly different from ‘Plg’ condition (*P*<0.05). (**i**) Representative confocal images of Plg^647^ (25 nm, red) uptake in control astrocytes cultures or in astrocytes cultures pre-treated with Pln (50 nM) or with Pln and aprotinin (20 IU ml^−1^) for 1 h; *n*=4). (**j**) Corresponding quantification of density (number of vesicles/10^3^ μm^3^) of fluorescent vesicles. Graphs show means±s.e.m. (*n*=4). *Significantly different from ‘control’ condition (*P*<0.05). Scale bars: 20 μm.

Next, we asked whether, as is the case with plasminogen, astrocytes are able to endocytose exogenous plasmin. To study the different forms internalized, we first generated fluorescent plasminogen and plasmin (Plg^488^, Plg^647^ and Pln^647^) and checked, by electrophoresis, that fluorescent plasminogen could be activated by tPA (Figure 4a). Then, coincubation of these fluorescent forms of plasminogen and plasmin on astrocytes revealed that plasmin, similar to plasminogen, was endocytosed in a time-dependent manner (Figure 4b and c). Plasmin-containing vesicles formed significantly faster than plasminogen-containing vesicles, which indicate a greater rate of endocytosis for plasmin (Figure 4d, 29.9±4.44 vesicles per min for plasmin vs 18.8±1.92 vesicles per min for plasminogen).

To address whether plasminogen needs to be activated into plasmin for its uptake, Pln^647^ or Plg^488^, in the presence or absence of tPA, were incubated on cultured astrocytes for 1 h before removal of extracellular proteins by a specific washing protocol [9]. Intracellular proteins were then extracted and submitted to sodium dodecyl sulphate polyacrylamide gel electrophoresis (SDS-PAGE). Analysis of fluorescence in the gel showed that when Plg^488^ is incubated alone, it is endocytosed in the form of plasminogen. Similarly, Pln^647^ is found inside astrocytes in the form of plasmin when incubated alone. Finally, when Plg^488^ is incubated in the presence of tPA, both forms (plasminogen and plasmin) are found within astrocytes (Figure 4e). Taken together, these data suggest that astrocytes can uptake both plasminogen and plasmin, and that plasminogen is not necessarily converted to plasmin before its uptake.

Because the main difference between plasminogen and plasmin relates to proteolytic activity, we then hypothesized that the difference in endocytosis rate between plasminogen and plasmin was linked to plasmin activity. To test this hypothesis, we incubated astrocytes with Plg^647^ alone or in combination with tPA to induce its conversion into plasmin, and measured the density of fluorescent endocytosis vesicles (Figure 4f and g). As expected, the coincubation of Plg^647^ with tPA led to the generation of plasmin (Figure 4h), and resulted in the detection of plasmin activity at the surface of astrocytes (Figure 4f). Interestingly, in these conditions, the number of fluorescent endocytosis vesicles was much higher than when Plg^647^ was incubated in the absence of tPA (Figure 4f and g). This increase in uptake was reversed by the addition of the inhibitor of plasmin activity, aprotinin (Figure 4f and g). These data indicate that the conversion of plasminogen into plasmin increases its uptake by astrocytes.

To explain this, we then hypothesized that a plasmin substrate was involved in the endocytosis of plasminogen and plasmin. We thus pre-treated astrocytes with plasmin, and extensively washed them, before adding Plg^647^ to the medium. In these conditions, plasminogen endocytosis was greatly enhanced (Figure 4i and j), and this effect was reversed by aprotinin pre-treatment (Figure 4i and j). These data show that plasmin activity stimulates plasminogen and plasmin endocytosis by acting on a plasmin substrate present at the surface of astrocytes.

### Cell-surface actin triggers plasminogen and plasmin uptake by astrocytes

**Figure 5:**
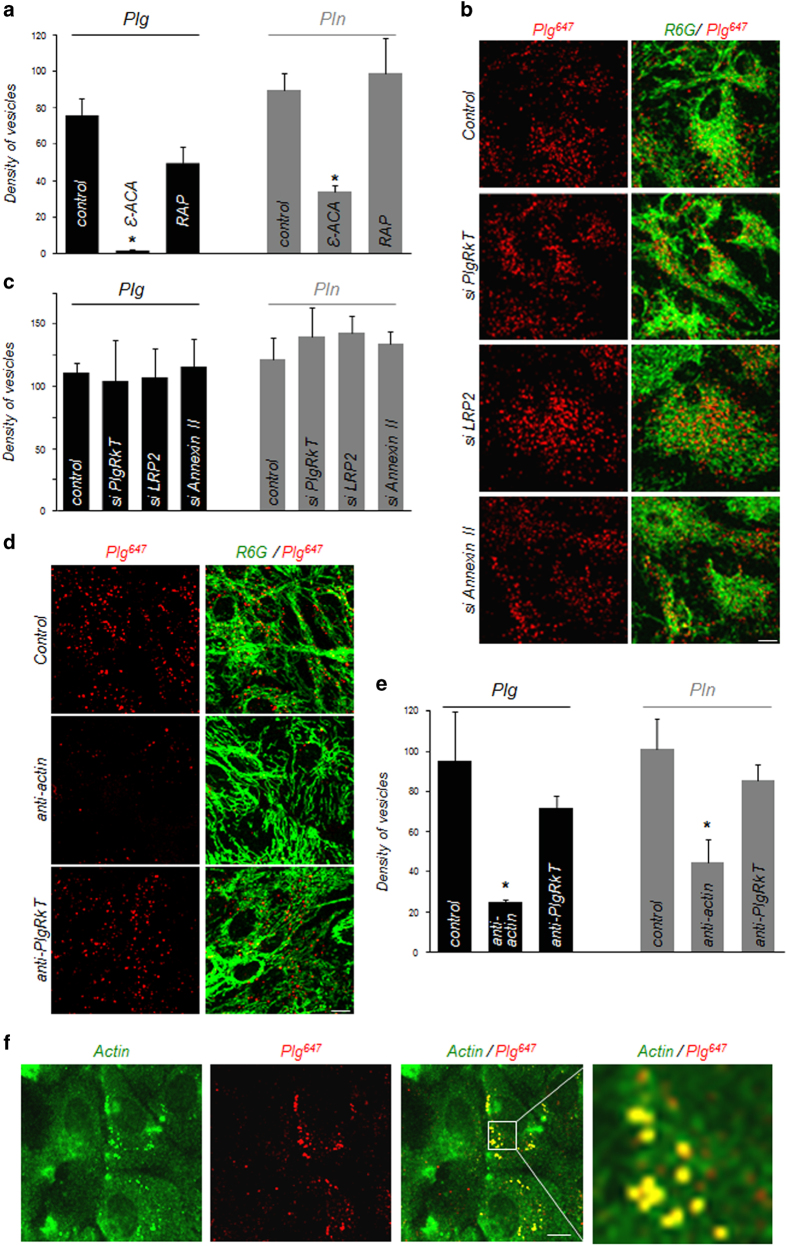
Cell-surface actin is involved in plasminogen (Plg) and plasmin (Pln) uptake. As Plg and Pln do not have the same rate of endocytosis, Plg was incubated for 2 h and Pln for 1 h in the following experiments. (**a**) Quantification of density (number of vesicles/10^3 ^μm^3^) of Plg^647^- (25 nm, for 2 h, black bars) and Pln^647^- (25 nm, for 1 h, grey bars) positive vesicles in control astrocytes or in astrocytes treated with ε-ACA (200 mm) or RAP (0.5 μm). Graphs show means±s.e.m. (*n*=4). *Significantly different from ‘control’ condition (*P*<0.05). (**b**) Representative confocal images of Plg^647^ (25 nm, red, 2 h) uptake in astrocytes (R6G, green) transfected with random siRNAs (control) or with siRNA directed against Plg-R_KT_, LRP-2 or Annexin II (*n*=5). (**c**) Corresponding quantification of density (number of vesicles/10^3 ^μm^3^) of fluorescent vesicles for Plg (black bars) and Pln (grey bars) (*n*=5). (**d**) Representative confocal images of Plg^647^ (25 nm, red) uptake in astrocytes (R6G, green) coincubated with antibodies against actin (25 nm) or Plg-R_KT_ (170 nm) (*n*=4). (**e**) Corresponding quantification of density (number of vesicles/10^3^ μm^3^) of fluorescent vesicles for Plg (incubated for 2 h, black bars) and Pln (incubated for 1 h, grey bars) (*n*=4). Data show means±s.e.m. *Significantly different from ‘control’ condition (*P*<0.05). (**f**) Representative photomicrograph of cell-surface actin immunocytochemistry (actin, green) in cultured astrocytes treated with Plg^647^ (25 nm, red) for 2 h. Scale bars: 20 μm.

Our next step was to identify the molecular target of plasmin present at the surface of astrocytes and responsible for plasminogen and plasmin endocytosis. As these molecules are internalized by a clathrin-dependent process, we hypothesized that a cell-surface receptor was necessary to drive endocytosis. Plasminogen receptors can be divided into three classes [10]: proteins synthesized with C-terminal basic residues similar to S100A10 within the Annexin II heterotetramer [11] or Plg-R_KT_ [12], proteins requiring a proteolytic processing to reveal a C-terminal basic residue, such as cell-surface actin [13] and proteins that bind plasminogen independently of basic residues exposure similar to LRP-2 (low density lipoprotein receptor-related protein 2) [14]. To identify the participating receptor, we first used a broad pharmacological approach targeting C-terminal basic residues with the lysine analogue ε-aminocaproic acid (ε-ACA) and targeting proteins from the LRP family, involved in endocytosis in other cell types [15] with the LRP antagonist RAP (receptor-associated protein). This enabled us to observe that plasminogen and plasmin internalization is significantly inhibited by ε-ACA but not by RAP, suggesting that the plasmin substrate involved in plasminogen and plasmin endocytosis bears a C-terminal basic residue (Figure 5a).

We next used small interfering RNAs (siRNAs) to knock down the expression of known plasminogen receptors in astrocytes. Knocking down of Plg-R_KT_, LRP-2 or Annexin II did not influence plasminogen or plasmin uptake (Figure 5b and c and Supplementary Figure S1A and B).

Another putative receptor for the binding of plasminogen and plasmin on astrocytes is cell-surface actin, previously shown to bind plasminogen on PC-12 cells and bovine chromaffin cells [13]. In addition, cell-surface actin has been described as a plasmin substrate: specific cleavage of this protein by plasmin reveals a free lysine and increases plasminogen and plasmin binding capacity [13]. Although knocking down actin expression would interfere with cell survival and can therefore not be used, the interaction of cell-surface actin with its cell-surface partners can be prevented by an anti-actin antibody [16]. Here, we observed that the coincubation with a polyclonal anti-actin antibody significantly inhibited plasminogen (Figure 5d and e) and plasmin (Figure 5e and Supplementary Figure S1C) endocytosis. Plasminogen and plasmin endocytosis were also inhibited by a monoclonal anti-actin antibody targeting the C-terminal end of actin (Supplementary Figure S1D and E). Besides, the use of a blocking antibody against Plg-RKT [12] did not significantly affect plasminogen/plasmin endocytosis (Figure 5d and e and Supplementary Figure S1C). Immunocytochemistry against cell-surface actin highlighted the colocalization of cell-surface actin with plasminogen-containing vesicles (Figure 5f). We next compared immunostaining in permeabilized and non-permeabilized astrocytes (Supplementary Figure S1F). In permeabilized astrocytes we observed an intracellular, fibrillar staining, corresponding to cytoskeleton-associated actin. In contrast, in non-permeabilized astrocytes, we observed a weaker, more diffuse cell-surface-associated staining corresponding to cell-surface actin. The absence of labelling in the absence of primary anti-actin antibody confirmed the specificity of these stainings (Supplementary Figure S1F).

Taken together, these data show that cell-surface actin is a molecular target of plasmin at the surface of astrocytes and participates in plasminogen and plasmin endocytosis.

### Plasminogen and plasmin are degraded through the lysosomal pathway after their uptake

**Figure 6:**
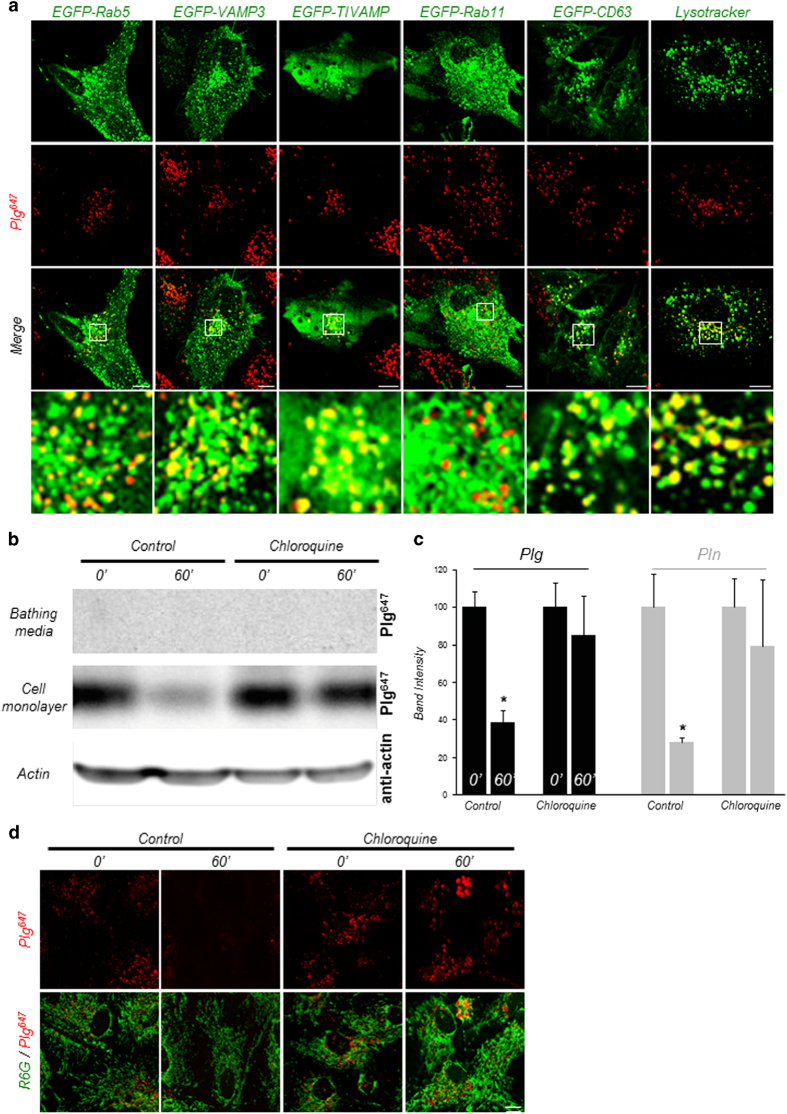
Plasminogen (Plg) and plasmin (Pln) are targeted to the degradation pathway after endocytosis. (**a**) Representative confocal images of cultured astrocytes transfected with pEGFP-Rab5, pEGFP-TI-VAMP, pEGFP-VAMP3 or pEGFP-CD63 vectors (top, green) show colocalization of Plg^647^ (25 nm, for 2 h, middle, red) and Rab5, TI-VAMP, VAMP3, CD63 but not with Rab11. Coincubation of LysoTracker (50 nm, green) and Plg^647^ (25 nm red) for 2 h shows lysosomal localization of Plg (*n*=3). (**b**) SDS-PAGE analysis of Plg^647^ content in the cell monolayer and in the bathing media of cultured astrocytes subjected to follow-up experiments after 2 h Plg^647^ (25 nm) loading (0 min) and after 1 h follow-up time (60 min) in the absence or in the presence of chloroquine (10 μM) applied during follow-up time (*n*=6). Plg^647^ content in the bathing media and in the cell monolayers was directly analysed after SDS-PAGE into the gels then a western blot was performed on the gel containing cell monolayers proteins to reveal cell-associated actin as a loading control with a polyclonal anti-actin antibody (1:1 000th). (**c**) Corresponding quantification of band densitometry for Plg (black bars) and Pln (grey bars, in Supplementary Figure 2B) (*n*=6). Histograms show means±s.e.m. *Significantly different from ‘0' condition (*P*<0.05). (**d**) Representative confocal images of cultured astrocytes subjected to follow-up experiments as described above (**c**) in the absence or in the presence of chloroquine (10 μm) (*n*=3). Scale bars: 10 μm.

Following their endocytosis, internalized molecules are routed to vesicular compartments through intracellular trafficking pathways. These pathways define the fate of internalized molecules by directing them to recycling or to lysosomal degradation pathways. Thus, to further investigate the intracellular traffic of plasminogen and plasmin, cultured astrocytes were transfected with a set of plasmids encoding enhanced green fluorescent protein (EGFP)-labelled markers of several types of trafficking vesicles, including Rab5 (a small GTPase localized in early endosomes), VAMP3 (vesicle-associated membrane protein 3), TI-VAMP/VAMP7 (tetanus neurotoxin-insensitive vesicle-associated membrane protein), CD63 (a late-endosomal/lysosomal marker) and Rab11 (a marker of recycling compartments) before incubation with fluorescent plasminogen (Plg^647^; Figure 6a) or fluorescent plasmin (Pln^647^; Supplementary Figure S2A). Exogenously supplied fluorescent plasminogen and plasmin colocalized with intracellular markers Rab5, VAMP3, TI-VAMP and CD63, but not with Rab11 (Figure 6a and Supplementary Figure S2A), suggesting a traffic of plasminogen and plasmin through early endosomes, late endosomes and lysosomal compartments within astrocytes, but not through recycling compartments. Colocalization with the lysosome marker lysotracker suggested that internalized plasminogen and plasmin are driven to the degradation pathway (Figure 6a and Supplementary Figure S2A).

To confirm the targeting of plasminogen and plasmin to the degradation pathway, we performed follow-up experiments in which fluorescent plasminogen or plasmin were added in the bathing media of astrocytes for 1 h at 37 °C (‘loading’ period), followed by extensive washing. Proteins were extracted from cell layers and bathing media after 1 h of incubation in fresh medium at 37 °C (‘follow-up’ period). SDS-PAGE electrophoresis of proteins extracted from the cell layer showed a disappearance of plasminogen and plasmin within astrocytes over follow-up time (Figure 6b and c). No increase in plasminogen or plasmin was detected in the corresponding bathing media during this period (Figure 6b and c), which suggests that plasminogen and plasmin are not recycled after uptake. Inhibition of the lysosomal pathway of protein degradation with chloroquine blocked the intracellular disappearance of plasminogen and plasmin during the follow-up time (Figure 6b and c and Supplementary Figure S3 for whole immunoblot). We confirmed these results with confocal imaging by applying the same follow-up experiment. After 60 min of follow-up time, we observed a disappearance of intracellular fluorescent plasminogen (Plg^647^; Figure 6d) or plasmin (Supplementary Figure S2B), a phenomenon blocked by chloroquine. These data show that, after their uptake by astrocytes, plasminogen and plasmin are targeted to the lysosomal pathway for degradation.

## Discussion

Taken together, the data obtained in this study suggest that astrocytes regulate the balance between plasminogen activation by tPA and clearance of plasmin. On the one hand, astrocytes promote the generation of plasmin by providing a surface for plasminogen activation and act as cofactors for plasmin proteolytic processing of its substrates. On the other hand, astrocytes trigger a negative feedback loop leading to endocytosis and degradation of plasminogen and plasmin. The increase in plasmin activity promotes the cleavage of cell-surface actin by plasmin, to release a free lysine. This lysine-bearing form of cell-surface actin can thus bind plasminogen and plasmin, as a necessary step for their endocytosis, finally leading to degradation. We thus propose here a fine mechanism for the regulation of plasmin activity at the cell surface of astrocytes: cell-surface actin, by the virtue of its sensitivity to plasmin activity, could sense this activity at the cell surface and drive plasminogen and plasmin clearance when needed to avoid excessive extracellular plasmin activity.

Although plasminogen activation by tPA at the surface of circulating fibrin is a well-characterized process involved in vascular fibrinolysis, little is known about the mechanisms driving plasminogen activation in the central nervous system. Extravascular plasminogen activation is commonly based on the interaction of tPA with plasminogen at the cell surface through specific binding of these proteins to specific receptors at the cell surface. Our experiments show that astrocytes highly stimulate tPA affinity for plasminogen and that resulting plasmin activity is localized at the surface of astrocytes, suggesting that astrocytes serve as a surface for plasminogen activation. In contrast, astrocytes do not stimulate uPA-induced plasminogen activation, which suggests that the stimulation of tPA-induced plasminogen activation is due to the expression of specific receptors for both tPA and plasminogen on astrocytes. We report here that this action is not mediated by any of the classical receptors for plasminogen and/or tPA (LRP-1, LRP-2, Annexin II or Plg-R_KT_).

It was reported earlier that injured, dying and dead cells in culture can promote plasminogen activation by tPA [4]. Here we observed that, although a low proportion of dead cells contribute to plasminogen activation—which confirms these previous observations [4]—the vast majority of plasmin activity in our cultures is found at the surface of living cells (Supplementary Figure S4). We thus describe here a novel mechanism of promotion of plasminogen activation by living astrocytes, which adds to the previously described processes in dead or dying cells. Because more than 90% of astrocytes are living cells in our cultures (data not shown), the promotion of plasminogen activation described here is mostly due to living cells.

Astrocytes are not the only cell type able to promote plasminogen activation in the brain: neurons also contribute to plasmin formation [17]. The relative importance of neurons and astrocytes in this process is difficult to establish, but we can postulate that whereas the two systems can coexist in the grey matter, astrocytic plasminogen is likely to be predominant in the white matter, where neuronal membranes are mostly covered by myelin, and can therefore not serve as a surface for plasminogen activation.

Localized plasminogen activation at the surface of astrocytes could have important implications in cerebral physiology. Within synapses, extracellular conversion of pro-BDNF into its mature form, mBDNF, by plasmin stimulates synaptic plasticity [1]. We report here that astrocytes are able to stimulate highly pro-BDNF activation by facilitating plasmin generation. This process could reveal a regulated dialogue between neurons, secreting inactive proteins (plasminogen, pro-BDNF) and astrocytes, stimulating their activation to allow them to feedback on neurons.

We also show here that astrocytes promote extracellular fibrinolysis, which is particularly relevant to multiple sclerosis and its animal models, in which intracerebral fibrinogen/fibrin deposition drives deleterious effects [18]. Interestingly, most of the cerebral lesions are characterized by astrocyte proliferation, a phenomenon called ‘astrogliosis’. As these astrocytes are subjected to phenotypic changes, it would be interesting to investigate how astrocyte activation influences plasminogen activation and resulting fibrinolysis.

Because excessive extracellular plasmin activity can promote deleterious effects in the brain, contributing notably to neuronal death [19], inflammatory processes [20] and blood–brain barrier opening [21], an efficient regulation system is necessary. Regulation of tPA in the central nervous system relies on inhibition of proteolytic activity by serine protease inhibitors (PAI-1, neuroserpin, protease nexin-1) [22] and on regulated internalization of tPA by astrocytes [7]. The present work demonstrates that astrocytes are also able to promote plasminogen activation and plasminogen/plasmin clearance. Astrocytes thus constitute a crossroad for the plasminogen activation system by promoting different levels of regulation: (i) promotion of plasminogen conversion, (ii) production of serine protease inhibitors, (iii) uptake of tPA for subsequent recycling and (iv) uptake of plasmin for its clearance. We report that astrocytes take up plasmin at a higher rate than plasminogen. Thus, the regulated endocytosis mechanism described here could enable an optimal control of plasmin concentration: under conditions of low extracellular plasmin activity, the low efficiency of uptake mechanisms could contribute to maintain basal, physiological cerebral plasmin activity. In contrast, in case of a significant rise of cerebral plasmin activity, adaptation of uptake mechanisms could support the removal of extracellular plasminogen and plasmin, to avoid toxic effects of excessive plasmin activity.

The regulation of the plasminogen activation system by astrocytes could have implications in a broad range of pathological situations. In Alzheimer’s disease, astrocytes have been shown to participate in extracellular amyloid-β peptide clearance [23] and, in parallel, plasmin has been shown to degrade amyloid-β peptide [24]. In light of the present study, further studies should address whether the regulation of plasminogen activation by astrocytes could participate in amyloid-β clearance and/or degradation. Finally, plasmin activation at the surface of astrocytes can directly induce astrocyte responses. For instance, plasmin can activate intracellular signalling pathways in astrocytes, leading to the retraction of astrocyte end-feet, a process considered to regulate blood–brain barrier function [25], which has implications in many physiological and pathological situations [26].

Most of the known receptors for plasminogen and plasmin are generally described to interact with the two forms with the same affinity [10] via the binding of their kringle domains to basic amino acids, particularly C-terminal lysine, exposed by receptors. However, in some cases, plasmin(ogen) receptors require cleavage by proteases like plasmin to expose their C-terminal lysin. This mechanism has been demonstrated for actin exposed at the surface of cells [13]. Here we report that among the yet described plasmin(ogen) receptors, only cell-surface actin is involved in plasminogen and plasmin uptake by astrocytes. This is consistent with the fact that plasmin activity stimulates plasminogen and plasmin internalization: extracellular plasmin activity can lead to the processing of cell-surface actin, thus revealing plasminogen and plasmin binding sites to enable their internalization and their clearance from the extracellular space. In this way, cell-surface actin could serve as a sensor of excessive extracellular plasmin activity.

Cell-surface actin has not been described before as an endocytosis receptor. Nevertheless, we report here that the blockade of cell-surface actin leads to the reduction of plasminogen and plasmin endocytosis. Thus, we conclude that the binding of plasmin(ogen) to cell-surface actin is a necessary step for plasmin(ogen) uptake, but we cannot assert whether cell-surface actin is the endocytosis receptor for plasmin(ogen) or if it has an indirect role by presenting plasminogen and plasmin to a yet unidentified endocytosis receptor.

The fact that, under physiological conditions, plasminogen is mainly expressed by neurons [27, 28], within secretory vesicles of neuronal processes [28], suggests a neuromodulatory role for plasminogen and plasmin, which can influence synaptic activity and plasticity by promoting maturation of proneurotrophins. The present study gives clues to understand how these neuromodulatory actions of plasmin(ogen) are regulated by astrocytes within the tripartite synapse.

Earlier studies have shown that astrocytes, in addition to neurons, can express active tPA [29]. In the present work, we observed that the addition of exogenous plasminogen to cultured astrocytes, in the absence of exogenous tPA, resulted in measurable plasmin activity (Supplementary Figure S5), although this activity was ~10-fold inferior to what measured in the presence of exogenous tPA (10 nm; Supplementary Figure S5). This indicates that astrocytes produce low amounts of active tPA (below the limit of detection corresponding to 1 nm; data not shown), which participate, although in low proportion, in the generation of plasmin activity from exogenous plasminogen.

The question as to whether the processes described here in cultured astrocytes also exist *in vivo* is crucial. Also, the possibility exists that astrocytes could promote plasmin(ogen) uptake and/or plasminogen activation with a different efficacy depending on their region of origin within the central nervous system, or on their function (blood–brain barrier function, tripartite synapse modulation or central nervous system repair, to name a few). Finally, pathological conditions within the central nervous system may also modify the ability of astrocytes to uptake plasmin(ogen) and/or activate plasminogen. These exciting questions should be the purpose of further studies in appropriate animal models.

The regulation of plasminogen activation by astrocytes may have important repercussions in pathological conditions, and notably in ischaemic stroke. In this pathology, crossing of tPA and plasminogen through the opened blood–brain barrier, as well as overexpression of the plasminogen activation system, contribute to excitotoxic neuronal death [30, 31]. In these conditions, astrocytes, as a core part of the neurovascular unit, could support neuronal protection by internalizing tPA and plasmin.

## Materials and Methods

### Reagents

Recombinant human Glu-Plg and recombinant human Pln were purchased from Enzymes Research Laboratories (South Bend, IN, USA). Recombinant human tPA (Actilyse) was purchased from Boehringer Ingelheim (Ingelheim am Rhein, Germany). Recombinant human uPA (Actosolv Urokinase) was purchased from Eumedica (Manage, Belgium). Alexa Fluor 488 and 647 carboxylic acid succinimidyl ester, fibrinogen from human plasma, Alexa Fluor 647 conjugate, CO_2_-independent medium, LysoTracker Green DND-26, fetal bovine serum and horse serum were purchased from Life Technologies (Manage, Belgium). Dulbecco’s modified Eagle’s medium (DMEM), DMEM-F12, dimethyl sulfoxide, Tween-80, phosphate-buffered saline (PBS), paraformaldehyde, l-glutamine, dynasore hydrate, monodansylcadaverine, ε-ACA, chloroquine diphosphate salt, R6G, propidium iodide, DAPI (4',6-diamidino-2-phenylindole) hydrochloride, TRI reagent, albumin from human serum, and rabbit polyclonal antibody and mouse monoclonal antibody (clone AC-40) were purchased from Sigma-Aldrich (St Louis, MO, USA). Recombinant human pro-BDNF was purchased from Novoprotein (Summit, NJ, USA). Aprotinin (Trasylol) was purchased from Bayer (Leverkusen, Germany). Receptor-associated protein (RAP) was purchased from Gentaur (Kampenhout, Belgium). Collagen I from rat tail was purchased from BD Biosciences (Le Pont de Claix, France). SYBR Green Supermix was purchased from Bio-Rad (Hercules, CA, USA). Anti-Plg-R_KT_ monoclonal antibody mAb 7H1 was used to block Plg-R_KT_ [12].

### Cell culture

Cortical astrocyte cell cultures were prepared from 1 to 3 days postnatal mice. Cerebral cortices were dissected and dissociated in DMEM. Then, cells were plated in DMEM supplemented with 10% fetal bovine serum, 10% horse serum and 2 mm glutamine on collagen I (0.05 mg ml^−1^)-coated plates. The medium was changed two times weekly. Experiments were performed when cells reached confluency, after 7–9 days *in vitro*.

Mixed cortical cultures containing both neuronal and glial cells were prepared from fetal mice at 15–16 days. Cerebral cortices were dissected and dissociated in DMEM. Dissociated cells were plated in DMEM supplemented with 5% fetal bovine serum, 5% horse serum and 2 mm glutamine at a density of about 3×10^5^ cells per well on an established bed of glia prepared as described above. Experiments were performed after 7–8 days *in vitro*.

### Labelling of plasminogen, plasmin, albumin, fibrinogen and pro-BDNF with fluorescent Alexa

As Alexa Fluor succinimidyl esters react with primary amines, proteins (recombinant plasminogen, plasmin, albumin and pro-BDNF) were first dialysed at 4 °C overnight against sodium bicarbonate buffer (NaHCO_3_ 0.1 m, Tween-80 0.1%, pH 8.4) to remove non-proteinaceous molecules containing primary amines. Then, purified proteins were mixed with the *N*-succinimidyl ester of Alexa^488^ or Alexa^647^ for 4 h at 4 °C with continuous stirring. The resulting solution was dialysed in bicarbonate buffer overnight at 4 °C to remove unbound dyes. Afterwards, Alexa-labelled proteins were frozen and stored at −80 °C until further use.

### Plasminogen/plasmin internalization assay

Pure primary cultures of astrocytes or mixed cultures of astrocytes and neurons were first washed in CO_2_-independent medium containing R6G (10 nm). Then, plasminogen or plasmin were incubated at the doses and for the time periods indicated in the result and figures sections.

Some treatments were realized on astrocytes: tPA (from 10 to 100 nm); uPA (1 UI ml^−1^, equivalent to tPA activity at 25 nm); aprotinin (dose 20 UI ml^−1^).

### Immunocytochemistry

Astrocyte cultures were washed with PBS, fixed in paraformaldehyde 4% for 20 min at room temperature, washed in PBS (0.1 m) and blocked 1 h in PBS containing albumin (4%). The following primary antibodies were used: mouse anti-glial fibrillary acidic protein monoclonal antibody (dilution 1:3 000; Merck Millipore, Billerica, MA, USA; MAB3402), chicken anti-MAP-2 polyclonal antibody (dilution 1:3 000; Abcam, Cambridge, UK; ab5392), rabbit anti-clathrin heavy-chain polyclonal antibody (1 μg ml^−1^; Abcam; ab21679), rabbit anti-actin polyclonal antibody (dilution 1:200; Sigma-Aldrich; A2066) and mouse anti-actin monoclonal antibody (dilution 1:1 000). Secondary fluorescent antibodies (dilution 1:600) were purchased from Jackson Immunoresearch (Bar Harbor, ME, USA). A counterstaining with DAPI (3 ng ml^−1^) was realized in some experiments. Confocal laser-scanning microscopy was performed using a Leica SP5 II confocal microscope (Leica, Wetzlar, Germany).

### Plasmin activity assays

Two different protocols were used to evaluate plasmin activity: an enzymatic assay to quantify plasmin activity and a confocal imaging based protocol to localize plasmin activity at the cell level.

### Enzymatic assay

Astrocyte cultures seeded in 96-well plates were serum-starved for 1 h with CO_2_-independent medium before treatment. Cleavage of the plasmin-specific chromogenic substrate S-2251 (0.3 mm; Chromogenix, Bedford, MA, USA) was followed by measuring variations in absorbance at 405 nm over time with an ELx800 Absorbance Reader (Bio-Tek, Winooski, VT, USA).

### Confocal imaging

Plasmin activity was imaged with a specific plasmin fluorescent substrate (Sensolyte AFC Plasmin Activity Assay Kit; Anaspec, Fremont, CA, USA; 10 μm). Living cultures were imaged using a Leica SP5 II confocal microscope.

### Protein extraction

Cells were lysed at 4 °C in TNT buffer (Tris-HCl, 50 mm; NaCl, 150 mm and 0.5% Triton X-100; pH 7.4) for 1 h. To clear lysates, samples were centrifuged for 15 min (13 000 *g*) at 4 °C. The amount of protein in each sample was measured by using BCA Protein Assay (Pierce, Waltham, MA, USA).

### SDS-PAGE

Fifteen micrograms of proteins were loaded in 10% polyacrylamide gel. Fluorescence of Plg^488^, Plg^647^, Pln^647^, fibrinogen^647^ and its degradation products (FDP) was directly visualized in a 10% polyacrylamide gel and fluorescence of pro-BDNF^488^ and mBDNF^488^ was directly visualised in a 15% polyacrylamide gel using ImageQuant LAS 4000 Camera (GE Healthcare, Chicago, IL, USA). Polyacrylamide gels were then transferred on a PVDF membrane and immunoblotting anti-actin (anti-actin, dilution 1:1 000; rabbit polyclonal antibody, dilution 1:1 000) was performed following a standard procedure. After incubation with the secondary antibodies, proteins were visualized with an enhanced chemiluminescence western blot detection reagent (GE Healthcare) using ImageQuant LAS 4000 Camera (GE Healthcare).

### Extraction of total RNAs

Total RNAs were extracted from cultured cells by using TRI reagent according to the manufacturer's instructions.

### Quantitative real-time PCR

Total RNAs (1 μg) from each sample were reverse transcribed using M-MLV reverse transcriptase (Invitrogen, Waltham, MA, USA). Primers were designed for each gene using ‘primer 3′ program (http://bioinfo.ut.ee/primer3-0.4.0/primer3/input.htm). Primer alignments were performed with the BLAST database to ensure the specificity of primers. The following primer sequences were used: GAPDH forward, 5′- TGCGACTTCCAACAGCAACTC-3′; GAPDH reverse, 5′- ATGTAGGCCATGAGGTCCAC-3′; Plg-R_KT_ forward, 5′- CCAAGTCCAAGAGAGCAAGG-3′; Plg-R_KT_ reverse, 5′- ATGGTCAGATGCCTTTCAG-3′; LRP-2 forward, 5′- CGTGGCCAGATTTCCTATGC-3′; LRP-2 reverse, 5′- AGGCAATGCCATCAGTAACC-3′; Annexin II forward, 5′- CACCAACTTCGATGCTGAGA-3′; Annexin II reverse, 5′- CAAAATCACCGTCTCCAGGT-3′. PCR reagents were prepared with RNase-free water-containing primers and IQ SYBR Green Supermix. For PCR amplification, mix (20 μl) was added to reverse transcription reaction (5 μl) previously diluted (1:20). Two negative controls were performed during each quantitative PCR experiment: reactions without reverse transcription to confirm the absence of genomic DNA contamination, and samples with no added cDNA template to prove the absence of primer dimers. Assays were run in triplicate on the Chromo 4 Real-Time PCR Detector (Bio-Rad). Amplification conditions were as follows: Hot Goldstar enzyme activation, 95 °C for 3 min; 50 cycles of PCR (denaturation: 95 °C, 15 s and hybridation/extension 60 °C, 1 min). GAPDH was used as a housekeeping gene. The levels of expression of gene of interest were computed as follows: relative mRNA expression=2^−(Ct of gene of interest^^−Ct of gene of GAPDH)^, where Ct is the threshold cycle value.

### Silencing mRNA

Smart Pool siRNA against LRP-2, Annexin II and Plg-R_KT_ (30 nm; Dharmacon, Lafayette, CO, USA) were transfected into primary cultures of astrocytes using Lipofectamine 2 000 (3 μg ml^−1^) according to the manufacturer’s instructions. The Stealth RNA-negative Control Duplex (Invitrogen) was used as a control condition. Astrocytes were treated 48 h after transfection.

### cDNA transfection

pEGFP-TI-VAMP, pEGFP-VAMP3 and pEGFP-Rab11 (Thierry Galli, University Pierre and Marie Curie, Paris, France); pEGFP-Rab5 (kind gift from Letizia Lanzetti, University of Turin, Turin, Italy); pEGFP-CD63 (kind gift from John Paul Luzio, Cambridge University, Cambridge, UK).

Plasmids (1 μg ml^−1^) were transfected in cultures of astrocytes using Lipofectamine 2 000 (3 μg ml^−1^; Invitrogen) according to the manufacturer’s instructions. At 48 h after transfection, astrocytes were treated with Plg^647^ (25 nm) or Pln^488^ (25 nm) during 2 h and live cells were imaged using a Leica SP5 II microscope.

### Image analysis

Confocal images were analysed with the ImageJ software. Number of vesicles was measured in each field with ‘analyse particles’ function. Total volume of astrocytes cytoplasm was estimated with R6G staining.

### Follow-up assay

Confluent astrocytes cultures were serum starved for 1 h in DMEM-F12 and were then exposed to 50 nm of fluorescent plasminogen (Plg^647^) or plasmin (Pln^647^) (‘loading’). Then, cells were washed two times with PBS (0.1 m), once with mild acid wash buffer [9] to remove cell-bound proteins and two times with PBS (0.1 m) before replacing the medium with fresh DMEM-F12 with or without chloroquine (10 μm) to inhibit lysosomal protein degradation. After 1 h of incubation in the fresh medium (‘follow-up’), bathing media and protein extracts from cell monolayers were collected and subjected to SDS-PAGE (10%). Plg^647^ or Pln^647^ fluorescence was visualized using ImageQuant LAS 4000 Camera (GE Healthcare).

### Statistical analysis

Results are expressed as means±s.e.m. Statistical analyses were performed by the Kruskall–Wallis test, followed by *post hoc* comparisons with the Wilcoxon's test to compare related samples.
