# Supplementary material for: Astrocytes regulate the balance between plasminogen activation and plasmin clearance via cell-surface actin
Source: Cell Discov. 2017 Feb 21;3:17001–. doi: 10.1038/celldisc.2017.1 (PMC5318850; doi:10.1038/celldisc.2017.1)
Supplement: Supplementary Information [file celldisc20171-s1.pdf]

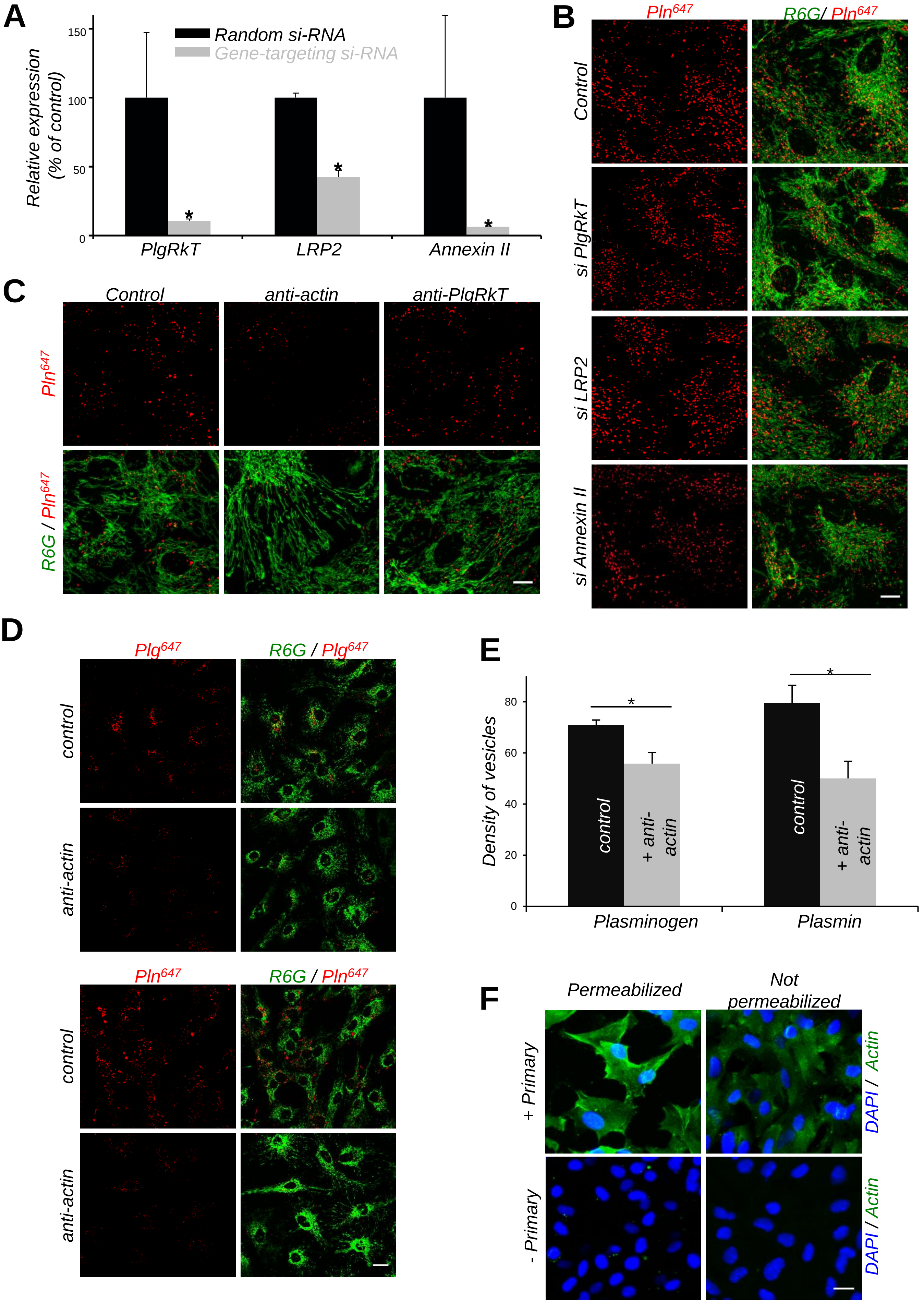

Suppl. Figure 1

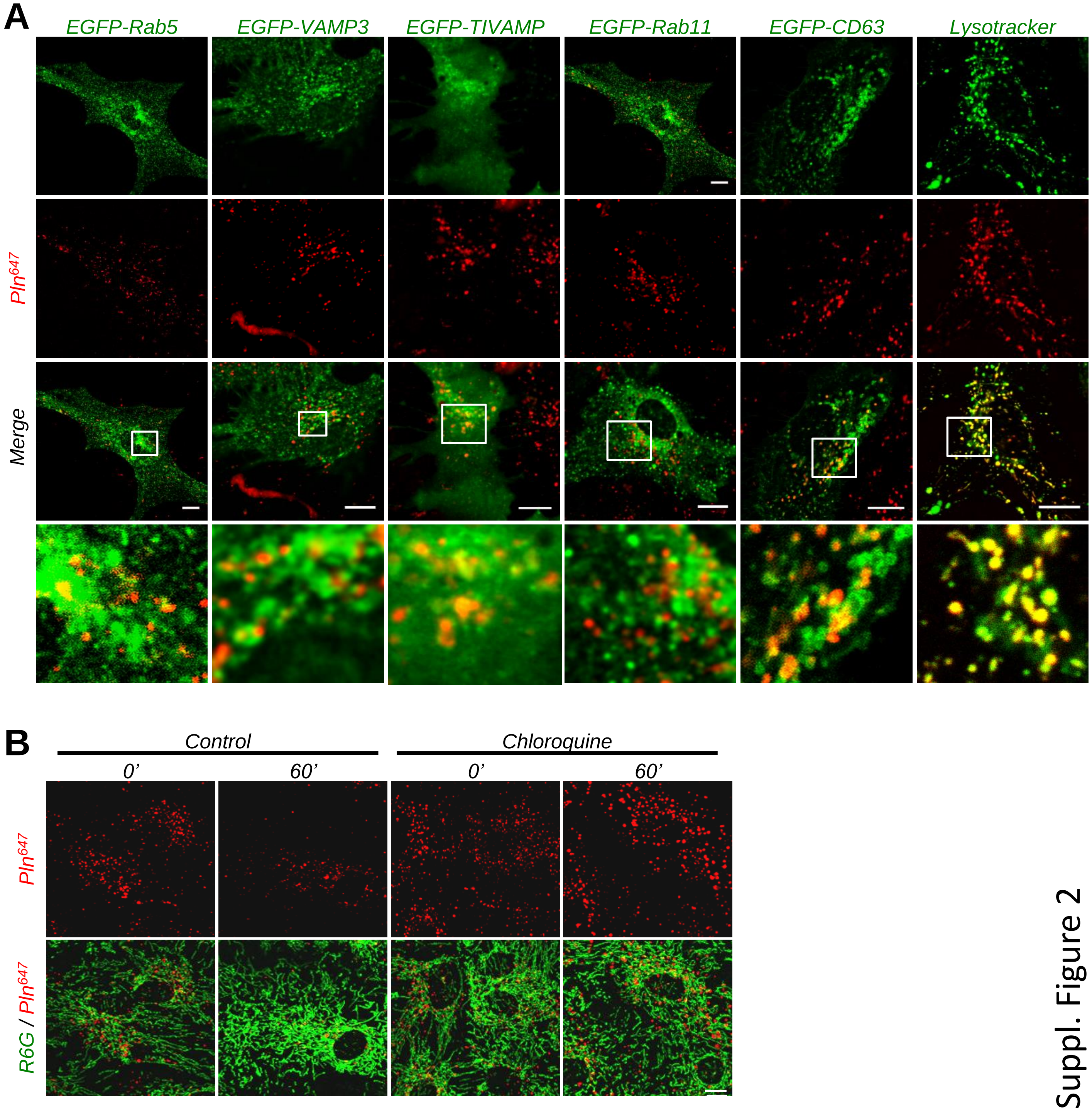

Suppl. Figure 2

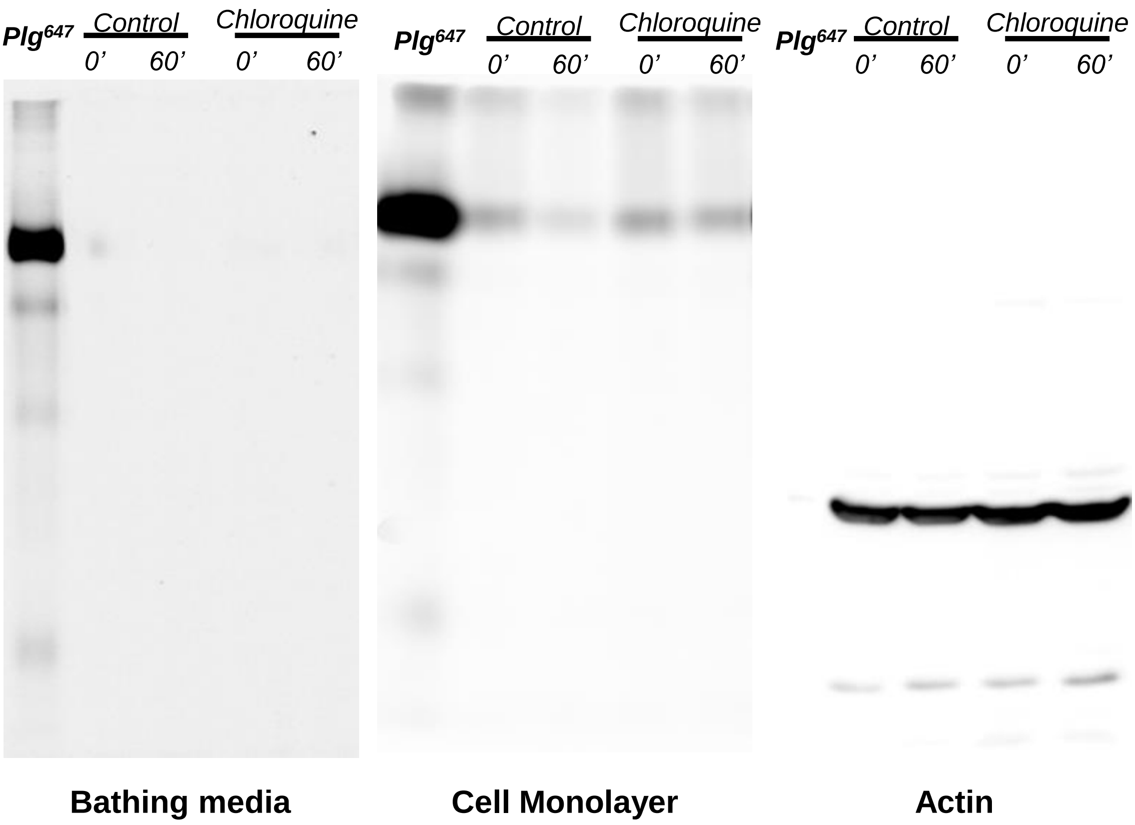

Suppl. Figure 3

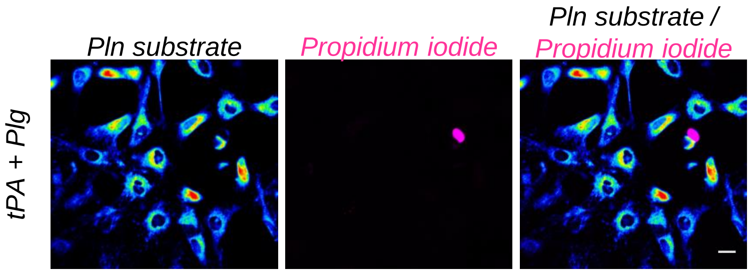

Suppl. Figure 4

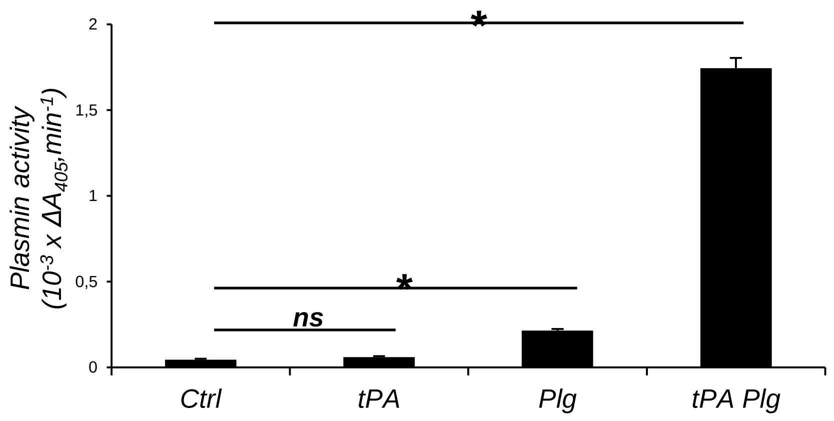

Suppl. Figure 5

Supplementary Figure 1. (**A**) Relative expression of $PlgR_{KT}$, LRP2 and Annexin II mRNA in astrocytes transfected with specific gene-targeting siRNAs (grey bars) or with random si-RNAs (black bars). Histograms show means ± SEM. *Significantly different from "control" condition (P<0.05). (**B**) Representative confocal images of Pln^{647} (25nM, red) uptake in astrocytes (R6G, green) transfected for 48 hours with random siRNAs (control) or with siRNA directed against Plg-R_{KT}, LRP-2 or Annexin II; n=5. (**C**) Representative confocal images of Pln^{647} (25nM, for 1h, red) and uptake in astrocytes (R6G, green) co-incubated with blocking antibodies against actin (25nM) or Plg-R_{KT} (170nM); n=4. (**D**) Representative confocal images of Plg^{647} (incubated for 2h, 25nM, red, top) or Pln^{647} (incubated for 1h, 25nM, red, bottom) uptake in astrocytes (R6G, green) co-incubated in the absence (*control*) or in the presence (*anti-actin*) of monoclonal anti-actin antibody (150 nM). (**E**) Corresponding quantification of density (number of vesicles/10^3 μm^3) of fluorescent vesicles for Plg (incubated for 2h, left) and Pln (incubated for 1h, right) in the absence (*control*, black bars) or in the presence (+ *anti-actin*, grey bars) of monoclonal anti-actin antibody ; n=3. Data show means ± SEM. *Significantly different from corresponding "control" condition (P<0.05). (**F**) Representative photomicrograph of permeabilized (left) or not permeabilized (right) cultured astrocytes labelled (+ *Primary, top*) with polyclonal anti-actin antibody (*Actin*, 1:40th, green) and DAPI. We checked specificity of the staining with a control without primary antibody (- *Primary*, bottom). Scale bars: 20 µm.

Supplementary Figure 2. (**A**) Representative confocal images of cultured astrocytes transfected during 48 hours with pEGFP-Rab5, pEGFP-TI-VAMP, pEGFP-VAMP3 or pEGFP-CD63 vectors (top, green) show colocalization of Pln^{647} (25 nM, 1h, middle, red) and Rab5, TI-VAMP, VAMP-3, CD63 but not with Rab11. Co-incubation of LysoTracker® (50 nM, green) and Pln^{647} (25nM for 1h, red) shows lysosomal localization of plasminogen (n=3). (**B**) Representative confocal images of cultured astrocytes subjected to follow-up experiments as described above (figure 6D) in the absence or in the presence of chloroquine (10 µM; n=3). Scale bars: 10 µm.

Supplementary Figure 3: Whole immunoblot corresponding to Figure 6B.

Supplementary figure 4: Representative photomicrographs of cultured astrocytes incubated with Propidium Iodide (1 µg/ml, pink) to label dead cells and co-incubated with recombinant tPA (25nM), plasminogen (50nM) and plasmin specific fluorescent substrate (Pln substrate) to reveal plasmin activity. Scale bars: 20 µm.

Supplementary Figure 5: Cultured astrocytes were incubated alone (Ctrl) or with recombinant tPA (tPA, 10nM), recombinant plasminogen (Plg, 25nM) or a combination of tPA and Plg (tPA/Plg, 10nM/25nM), and plasmin activity was measured with quantitative enzymatic assay.
